# Fluoroscopy-Guided Resolution of Ingested Thrombus Leading to Functional Disturbance of a Continuous-Flow Left Ventricular Assist Device

**DOI:** 10.1155/2012/791056

**Published:** 2012-10-10

**Authors:** Jens Garbade, Hartmuth B. Bittner, Friedrich-Wilhelm Mohr, Markus J. Barten

**Affiliations:** Department of Cardiac Surgery, Heart Center Leipzig, University of Leipzig, Struempellstra**β**e 39, 04289 Leipzig, Germany

## Abstract

The third generation of left ventricular assist devices (LVADs) has been shown to improve outcome and quality of life in patients suffering from acute and chronic heart failure. However, VAD-associated complications are still a challenge in the clinical practice. Here we report the resolution of a mobile thrombus formation in the proximity of the inflow cannula of a third generation of LVADs (HVAD Pump, HeartWare, Inc.) in a patient with chronic heart failure 4 months after implantation.

## 1. Introduction

The development of continuous-flow left ventricular assist devices evolved from an unaccepted, nonviable strategy to a proven and adopted treatment for patients with end-stage heart failure [1]. Despite this increased adoption, chronic support may have a higher rate of device-related thromboembolic complications and this is associated with significant morbidity and mortality [2, 3]. The event rates quoted for LVAD thromboembolism vary considerably from 1.1 to 34.8% between devices [4]. Therefore, patient adherence and education are mandatory to ensure sufficient anticoagulation according to the current recommendations. Severe pump thrombosis is a relatively rare complication and therapeutic experience is limited. The application of systemic thrombolytics with axial and centrifugal flow pumps has been described in the past [5, 6]. In this paper we describe the application of intraventricular thrombolytics in a third-generation centrifugal flow pump. The HVAD Pump (HeartWare, Inc.) is a miniaturized, continuous-flow device that is completely implanted within the pericardial space for uni- and possible biventricular support [7, 8]. The recommended target international-normalized ratio (INR) is 1.7 to 2.3 in addition to antiplatelet therapy.

Here we describe the resolution of a significant thrombus formation in the proximity of the inflow cannula of a HVAD^*™*^ Pump with intra-ventricular fluoroscopy-guided local lysis.

## 2. Case Presentation

A 60-year-old man (body weight 81 kg) was admitted to our institution for further heart failure therapy. However, the patient was eligible for transplantation and listed as high urgent. Due do recurrent incurable ventricular arrhythmias and catecholamine support an LVAD was implanted. The patient received an LVAD (HVAD Pump, HeartWare) continuous-flow pump. The postoperative course was uneventful and the patient was discharged 30 days after implantation. Four months later, the patient developed signs of heart failure and the controller displayed technical problems with the assist device.

**Figure 1:**
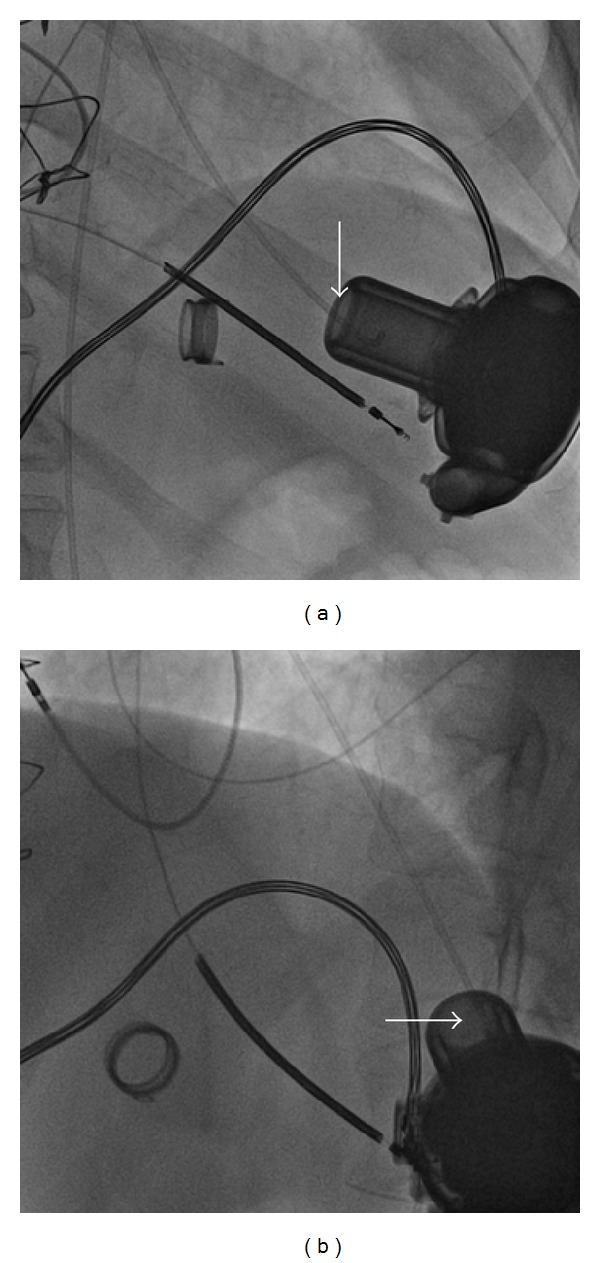
Imaging of fluoroscopy-guided thrombolysis. The 5-French pigtail catheter is placed directly in front of the LVAD inflow cannula (white arrows): (a) RAO exposure and (b) LAO exposure.

Clinically, the patient exhibited worsening shortness of breath and signs of hemolysis (dark red urine), and, additionally, an audible sound was detected from the pump. At the time of admission the patient's anticoagulation status was subtherapeutic (INR 1.3 and PTT 45 sec), so a heparin-bolus of 10.000 IE was given immediately, followed by continuous intravenous heparin administration to achieve a therapeutic PTT range of 80 to 100 sec. Initially, the pump speed was 2500 rpm with an estimated flow of up to 9 L/min and an abnormal increase in power consumption. Laboratory results showed evidence of severe hemolysis, for example, increase of bilirubin and LDH. Waveform downloads from the assist device, combined with reduced flow pattern and increased power requirement over time, suggested an obstruction of the inflow cannula.  Table 1 summarizes all relevant clinical- and device-associated data. Transthoracic echocardiography revealed reduced left ventricular function, moderate mitral regurgitation, intermittent aortic valve opening, and good right ventricular function. The septum had a normal position without any signs of suction suggesting a normal position of the left ventricle cannula. Additionally, the echo revealed a large thrombus formation obstructing the inflow cannula, resulting in partial occlusion. Subsequently, the patient was referred to our cath-lab for fluoroscopy-guided direct inflow cannula lysis. The LV was accessed via the right femoral artery using a 5-French pigtail catheter. The pigtail catheter was placed under fluoroscopic guidance directly into the proximity of the inflow-cannula. Additionally it was very important to avoid any ingestion of the catheter into the pump (Figure 1).Twenty-seven mg of alteplase (Actilyse) was administered at a rate of 1 mg/min under fluoroscopic guidance. Within 27 minutes of selective thrombolysis, energy consumption decreased to normal values from 15 to 4.0 watts and also the pump flow reached normal range (Table 1). The procedure was tolerated well without any bleeding complications. After removal of the catheter the femoral artery sealed with a closure device (AngioSeal), and the patient returned to the ICU.

The subsequent clinical course was uneventful and the pump continued to function well. After achieving an adequate anticoagulation status (INR 2 to 2.5) the patient was discharged home 5 days after intervention. 

## 3. Discussion

Assist device-associated complications are challenging problems in treating patients with mechanical circulatory support. The incidence of thrombus formation depends on anticoagulation status, type of mechanical support, and patient adherence [1]. Increased or sudden changes of pump parameters are evidence of potential complications. Despite the considerable advances in device technology, monitoring and management of the hypercoagulable status—resulting from foreign surfaces of the assist device system, altered rheologic conditions, and blood stasis in the patient— remain a challenge [4]. Anticoagulation with warfarin and often additionally with antiplatelet therapy exposes the patient to an increased risk of bleeding. This situation could exacerbate by alterations in kidney and liver function or by the nonadherence of the patient to follow the therapeutic anticoagulation recommendations. On the other hand, VAD-associated thromboembolic occurrences may also result in life-threatening complications. In case of severe VAD thrombosis leading to an acute irreversible pump stoppage a surgical intervention is required but often results in a high mortality and morbidity [2]. In milder case of VAD thrombosis medical or interventional therapy as systemic and local catheter-based thrombolysis has been reported as alternative approaches with variable outcome previously [4–6]. 

Here we described the resolution of a thrombus formation in the proximity of the inflow cannula of the HVAD continuous-flow assist device. Compared to open reexploration or systemic thrombolysis, the fluoroscopy-guided selective administration of fibrinolytic agents allows a dose reduction of these agents, and, therefore, to reduce the potential risk for general severe bleeding complications. 

Our paper indicates that the intracavitary lysis of thrombus-mediated inflow cannula obstruction of the HVAD pump may be an effective and safe concept. However, for out-hospital care and successful mechanical support, a stringent patient adherence, monitoring, and education are mandatory to avoid any complications, especially thromboembolic events.

## Figures and Tables

**Table 1:** HVAD parameters and laboratory data over the time (anticoagulation and hemolysis).

	Speed (RPM)	Flow (LPM)	Power (Watts)	INR	PTT (sec)	Total Bilirubin (mg/dL)	LDH (U/L)
Prior thrombus	2600	5.4	4	2.2	50	0.98	367
Admission	2600	up to 12.0	up to 15.0	1.3	45	3.53	4478
Heparinization	2500	up to 9.0	5.0–10.0	1.7	117	4.49	6420
LV-lysis	2500	4.0	4.0	1.5	139	3.56	5676
Discharge	2500	4.5	4.5	2.2	41	0.77	1194
Followup	2600	5.0	5.0	2.3	49	0.63	378

INR: international normalized ratio; PTT: partial thromboplastin time; LDH: lactate dehydrogenase.
